# Integrated analysis of microbiome and transcriptome reveals the mechanisms underlying the chlorogenic acid-mediated attenuation of oxidative stress and systemic inflammatory responses via gut-liver axis in post-peaking laying hens

**DOI:** 10.1186/s40104-025-01216-7

**Published:** 2025-06-06

**Authors:** Zhaoying Hu, Lianchi Wu, Yujie Lv, Chaoyue Ge, Xinyu Luo, Shenao Zhan, Weichen Huang, Xinyu Shen, Dongyou Yu, Bing Liu

**Affiliations:** 1 College of Animal Sciences, Zhejiang University, Hangzhou, 310058 China; 2 ZJU-Xinchang Joint Innovation Centre (TianMu Laboratory), Gaochuang Hi-Tech Park, Xinchang, 312500 China; 3 Hainan Institute, Zhejiang University, Sanya, 572000 China

**Keywords:** Chlorogenic acid, Gut-liver axis, Inflammation, Laying hen, Oxidative stress

## Abstract

**Background:**

Systemic inflammatory responses and oxidative stress occur in laying hens during the aging process, particularly during the post-peaking laying period, which generally result in multi-organ damages, leading to significant declines in egg performance and quality. Chlorogenic acid (CGA)-enriched extract from *Eucommia ulmoides* leaves has anti-inflammatory and antioxidant activities. However, the mechanisms underlying whether and how CGA alleviates systemic inflammatory responses and oxidative stress to improve egg performance and quality in post-peaking laying hens remain unclear. In this study, the potential regulatory mechanisms of CGA in alleviating inflammatory responses and oxidative stress along the gut-liver axis were investigated. A total of 360 55-week-old Hy-line white-laying hens were randomly selected and divided into four groups. The hens in the four groups were fed a basal diet (CON) or basal diets supplemented with 200, 400, and 800 mg/kg of CGA (CGA200, CGA400, and CGA800, respectively) for 10 weeks.

**Results:**

The results demonstrated that CGA significantly alleviated intestinal and hepatic damages resulting from systemic inflammatory responses and oxidative stress, thereby improving the laying performance and egg quality of post-peaking laying hens. CGA reduced systemic inflammation by improving intestinal barrier function and modulating inflammation-associated microbiota (*Blautia* and *Megamonas*), thus inhibiting endotoxin translocation. CGA can also reduce oxidative stress by upregulating the NRF-2 pathway-related genes and increasing antioxidant enzyme activities in the liver. The results of transcriptome sequencing revealed that CGA promoted lipid metabolism by regulating hepatic adipocytokine pathway-related genes/protein and reduced the inflammatory responses and apoptosis in liver by regulating PI3K/AKT pathway-related genes/proteins, which was also verified by qPCR and western blotting.

**Conclusion:**

CGA alleviated multi-organ damages and dysfunction by suppressing the systemic inflammatory responses and oxidative stress in post-peaking laying hens, thereby improving egg performance and quality. The optimal dose of CGA is 400 mg/kg in this experiment. These results provide a sound theoretical basis for the application of CGA as an exogenous animal feed additive for laying hens.

**Supplementary Information:**

The online version contains supplementary material available at 10.1186/s40104-025-01216-7.

## Introduction

Aging is a universal phenomenon associated with intrinsic factors such as chronic inflammation, oxidative stress, cellular senescence and gut microbial dysbiosis, which can lead to organ damages such as leaky gut and liver injury [1–3]. It has been shown that aging individuals exhibit significantly increased intestinal permeability and histological scores compared to young individuals [4]. Furthermore, aging-induced microbial dysbiosis disrupts the equilibrium between pro- and anti-inflammatory factors, thereby leading to systemic inflammation and age-related diseases [4, 5]. Aging also causes lipid over-deposition, cellular damage, and cell cycle arrest [3, 6]. Increasing studies show that gut microbiota involved in the onset and progression of liver diseases, including fatty liver hemorrhagic syndrome (FLHS) in laying hens and non-alcoholic fatty liver disease (NAFLD) in humans [7, 8]. Systemic inflammatory responses and oxidative stress often occur in laying hens during the aging process, especially during post-peaking laying period, which generally results in multi-organ damages along the gut-liver axis, leading to significant declines in egg performance and quality [9]. Thus, the gut-liver axis is one of the most important targets for improving the overall health and laying performance of laying hens.

Recent studies have demonstrated that dietary nutrients such as probiotics, polysaccharides, and plant extracts can modulate the gut-liver axis by balancing the redox state, modulating the gut microbiota, and reducing the inflammatory response, and thereby promoting gut and liver health and improving production performance in post-peaking laying hens [9–12]. Chlorogenic acid (CGA), a bioactive polyphenol, is the most abundant isomer of caffeoylquinic acid widely found in *Eucommia ulmoides*,* Lonicera japonica*, green coffee beans, and tea extracts [13]. Studies have demonstrated that CGA has multiple health-promoting properties such as anti-inflammatory, antioxidant, antimicrobial, and glycolipid metabolism regulation properties [13]. However, the intervention effects of CGA on oxidative stress and systemic inflammatory responses along the gut-liver axis in laying hens remain unclear. Gut microbiota is the primary driver of CGA digestion and absorption in the host [14]. Approximately one-third of CGA is absorbed in the stomach and small intestine into the bloodstream to reach the liver and other organs, whereas the remainder is absorbed and utilized by the gut microbiota [14]. In CCl_4_-induced rat and D-galactose-induced aging mouse models, CGA exhibited protective effects by restoring oxidative stress and inflammation in the liver and colon [15, 16]. In tamoxifen-treated rats, CGA significantly reduced serum myeloperoxidase activity, significantly decreased hepatic tumor necrosis factor-α (TNF-α) and interleukin (IL)-1β levels, and significantly increased hepatic IL-10 levels [17]. In addition, CGA ameliorated fatty acid metabolism disorders by modulating the AMP-activated protein kinase (AMPK)/acetyl-CoA carboxylase (ACC)/carnitine palmitoyl transferase 1 (CPT-1) signaling pathway [18].

In recent years, CGA has begun to be used as a feed additive in the livestock industry [13]. Studies have shown that CGA can improve intestinal morphology, barrier functions, and gut microbiota in weaned piglets and oxidative stress-challenged broilers [10, 19]. In addition, it was found to be conducive to the meat quality of broilers [20]. However, the precise effect of CGA on laying hens, especially on laying performance, egg quality, and gut-liver health in post-peaking laying hens, remains unclear. Therefore, in the present study, graded levels of CGA were added to post-peaking laying hens to investigate their effects on egg performance and quality. In addition, the potential regulatory mechanisms of CGA in alleviating systemic inflammatory responses and oxidative stress along the gut-liver axis were investigated using transcriptome and microbiome. The findings contribute to the comprehension of the latent mechanisms by which CGA improves the health of the gut and liver and provides supporting theories for promoting the application of CGA in poultry.

## Methods

### Experimental design

The whole experiment was conducted in strict accordance with the National Research Council’s Guide for the Care and Use of Laboratory Animals and authorized by the Animal Ethics Committee of Zhejiang University (approval No. ZJU20220310), and all experimental procedures and animal euthanasia were performed with full respect to animal welfare. In all, a total of 360 55-week-old Hy-Line White-laying hens with analogous initial egg production (88.50% ± 1.50%) and body weight (1.60 ± 0.16 kg) were acquired from a commercial farm. After a 1-week of adaptation, the hens were randomized divided into four groups, comprising 6 replicates in each group and 15 hens per replicate. Hens in the four groups were fed a basal diet (CON) or basal diets supplemented with 200, 400, and 800 mg/kg of CGA (CGA200, CGA400, and CGA800, respectively) for 10 weeks. CGA with 98% purity was purchased from Shaanxi Bolin Biotechnology Co., Ltd. (Xi’an, China). CGA was first mixed with the premix and then with other ingredients by step-by-step premixing. The formulation and nutrition levels of basal diet (Table 1) conformed to the recommended standards of National Research Council [21]. All hens were kept in regular cages with ad libitum feeding and appropriate environmental parameters (16 h/d light and 26 °C).

**Table 1:** Ingredients and nutrient contents of the basal diet (as fed-basis)

Ingredients	Content, %	Nutrient levels	Content^2^
Corn (8.0% CP)	57.00	Metabolizable energy, Mcal/kg	2.64
Soybean meal (46% CP)	24.00	Crude protein, %	16.33
Wheat middling	5.0	Lysine, %	0.81
Emulsified fat powder (50% Fat)	1.50	Methionine, %	0.45
Limestone	9.00	Cysteine + methionine, %	0.74
Dicalcium phosphate	1.00	Calcium, %	3.62
Salt	0.30	Total phosphorus, %	0.55
DL-Methionine	0.20	Available phosphorus, %	0.35
Premix^1^	2.00		
Total	100.00		

^1^The premix provided the following per kg of the diet: vitamin A, 12,500 IU; vitamin D_3_, 4,000 IU; vitamin K_3_, 2 mg; thiamine, 1 mg; riboflavin, 8.5 mg; calcium pantothenate, 50 mg; niacin acid, 32.5 mg; pyridoxine, 8 mg; folic acid, 5 mg; B_12_, 5 mg; choline chloride, 500 mg; iron, 60 mg; copper, 10 mg; manganese, 80 mg; zinc 80 mg; selenium 0.30 mg and iodine 0.30 mg

^2^All values were analyzed values excepted for the metabolizable energy and available phosphorus

### Sample collection

At the end of the feeding trial, 6 eggs from each replicate (a total of 36 eggs per group, *n* = 6) were randomly selected for egg quality analysis, and one hen from each replicate (*n* = 6) were randomly selected for tissue collection. The serum was obtained by centrifuging the wing vein blood samples at 3,000 × *g*, and then the obtained was immediately stored at −80 °C. All hens were euthanized and rapidly separated into the liver, small intestine (jejunum), and ovarian tissues. The liver, jejunum, and ovarian stroma were sectioned into small pieces and fixed in 4% paraformaldehyde, and another mid-jejunum sample was fixed in 2.5% glutaraldehyde. The remaining tissues and cecal contents were frozen for further analysis.

### Laying performance and egg quality

#### Determination of laying performance

For laying performance analysis, the number of eggs and total egg weight were monitored daily, and feed disappearance was recorded weekly on a replicate basis (*n* = 6) to calculate the laying rate (LR), average daily egg mass (ADEM), average daily feed intake (ADFI), and feed conversion ratio (FCR).

#### Determination of egg quality

At the end of the feeding trial, a total of 36 freshly laid eggs (6 eggs per replicate) were collected from each group (*n* = 6) for egg quality assessment within 24 h of collection. The egg shape index was determined using digital vernier calipers. The thickness of shell membrane-removed eggshell was measure by thickness gauge. The weights of egg/albumen/yolk, eggshell strength, yolk color, Haugh unit, and albumen height were determined by egg quality tester (DET-6000, Nabel Co., Ltd., Kyoto, Japan).

### Observation of tissue morphology and ultrastructure

#### Hematoxylin and eosin (H&E) staining

The fixed tissue samples (liver, jejunum, and ovarian stroma tissues) were embedded and then cut into slices. After H&E dyeing, the tissue section was observed and photographed under an optical microscope (Nikon Eclipse 80i microscope, Nikon, Japan). The jejunal villus height (VH) and crypt depth (CD) were determined by the ImageJ software processing.

#### Transmission electron microscopy (TEM)

The fixed jejunum tissues (approximately 1 mm^3^) were dehydrated using a graded ethanol series and sliced into sections. After uranyl acetate and lead citrate staining, the jejunal sections were subsequently obtained using TEM (JEOL-JEM-1200EX, Peabody, Massachusetts, USA), as previously described [22].

#### Oil Red O staining

The fixed hepatic tissues were embedded in the frozen section embedding agent, cut into frozen sections, and then dyed with Oil Red O. The sections were observed and photographed under an optical microscope (Nikon Eclipse 80i microscope, Nikon, Japan).

### TUNEL analysis

The TUNEL assay was performed using the apoptosis detection kit (Beyotime Biotechnology, Shanghai, China). After methanol fixation, antigen retrieval and TUNEL reagent incubation, the nuclei of the frozen liver sections were dyed with 4′,6-diamidino-2-phenylindole (DAPI). The sections were sealed with quenching sealer and then observed under a fluorescence microscope (BX-61, Olympus, Center Valley, Pennsylvania, USA).

### Serum hormones and lipopolysaccharide (LPS) content analysis

The follicle-stimulating hormone (FSH), luteinizing hormone (LH), estradiol (E2), progesterone (PROG), and LPS contents in serum were determined using chicken-specific kits (AF7321-A, AF4291-A, AF4341-A, AF4369-A, and AF81146-A, respectively; Aifang, Changsha, China).

### Oxidative stress parameters analysis

The activities of total antioxidant capacity (T-AOC), catalase (CAT), glutathione peroxidase (GSH-PX), and superoxide dismutase (SOD), and the content of malondialdehyde (MDA) in the supernatants of liver and jejunum homogenates, were determined using commercial kits (A015, A005, A007, A001, and A003, respectively; Nanjing Jiancheng Bioengineering Institute, China). The protein concentration in the supernatants of liver and jejunum homogenates was determined by bicinchoninic acid (BCA) method with the total protein assay kit (A045-3-2, Nanjing Jiancheng Bioengineering Institute, China). All oxidative parameter levels were normalized to the total protein content for comparison between samples.

### Inflammatory parameters analysis

The levels of interleukin-6 (IL-6), interleukin-10 (IL-10), tumor necrosis factor-α (TNF-α), and transforming growth factor-β (TGF-β) in the supernatant of liver and jejunum homogenates were determined by using respective broiler-specific ELISA kits (RK04981, RK04580, RK04961, and RK06255, respectively; ABclonal, Wuhan, China). The protein concentration in the supernatants of liver and jejunum homogenates was determined by BCA method with the total protein assay kit (A045-3-2, Nanjing Jiancheng Bioengineering Institute, China). All inflammatory parameter levels were normalized to the total protein content for comparison between samples.

### Eukaryotic reference transcriptome analysis

Based on the phenotype results, the liver samples from CGA400 and CON groups were selected for transcriptome analysis. Livers were homogenized and the total RNA was extracted using FreeZol Reagent (R711-01, Vazyme, Nanjing, China) according to the manufacturer’s instructions. RNA concentration was measured using a NanoDrop ND-1000 spectrophotometer (Nano-Drop Technologies, Wilmington, DE, USA). The purity of RNA (A_260_/A_280_) for all samples was above 1.80. The integrity of RNA was evaluated using the Agilent 2100 Bioanalyzer system (Agilent Technologies, Santa Clara, CA, USA). All samples had an RNA Integrity Number (RIN) value > 7. Then, the mRNA sequencing libraries were constructed and the mRNA libraries were sequenced on NovaSeq X Plus platform (PE150). Using transcripts per million reads (TPM) method to compute the expression levels of each transcript. Using DESeq2 to perform differential expression analysis. GO functional enrichment was performed using Goatools, and Kyoto Encyclopedia of Genes and Genomes (KEGG) pathway analysis was performed using Python software. All data visualizations were performed by R software.

### Validation of microarray data using quantitative real-time PCR (qPCR) analysis

The key differentially expressed genes (DEGs) were selected for qPCR analysis to verify the reliability of RNA sequencing results. RNA extraction and cDNA synthesization were conducted using commercial kits (R711 and R312, respectively; Vazyme, Nanjing, China). The qPCR was carried out by using a Real-Time PCR Detection Systems (CFX Connect™, Bio-Rad, Hercules, CA, USA) with Taq Pro Universal SYBR qPCR Master Mix (Q712; Vazyme, Nanjing, China). The mRNA expression levels of target genes were normalized by β-actin and were computed as 2^−ΔΔCt^. All primers were designed by NCBI Primer-BLAST tool and are listed in Table S1.

### Western blotting

To further verify the transcriptomic results, liver tissues of hens from CON and CGA400 groups were lysed using RIPA lysis buffer (Beyotime Biotechnology, Shanghai, China), and the total protein concentration was detected by BCA method with the total protein assay kit (Beyotime Biotechnology, Shanghai, China). Equal amounts of total protein were added to 12% SDS-PAGE for separation and transferred to nitrocellulose membranes (Millipore, MA, USA). The membranes were closed with 5% skimmed milk powder, followed by primary antibodies against β-actin (AC026, ABclonal, Wuhan, China), CPT-1 (PH5922S, Abmart, Shanghai, China), t-PI3K/p-PI3K (T40115/T40116, Abmart, Shanghai, China), and p-NF-κB/t-NF-κB (TP70621/T55034, Abmart, Shanghai, China) overnight at 4 °C with gentle shaking. Then, the membranes were washed and incubated with horse radish peroxidase-conjugated goat anti-rabbit-mouse universal antibody (HKI0029, HaoKeBio, Hangzhou, China) for 1 h at 37 °C. The protein bands were detected using Tanon 5200 detection systems (Tanon, Shanghai, China) and the relative band density was measured by ImageJ software using β-actin as the internal reference.

### 16S rRNA sequencing analysis

Based on the phenotype results, microbiota genomic DNA was extracted from the cecal contents of laying hens in the CON and CGA400 groups using TIANamp fecal DNA Kits (Tiangen Biotech, Beijing, China). The quality of the extracted genomic DNA was determined by 1% agarose gel electrophoresis, followed by determination of the DNA concentration and purity using a Nanodrop spectrophotometer (Thermo Scientific, Waltham, MA, USA). The 16S rDNA gene’s V3–V4 region was amplified using the primer pairs 338F and 806R and sequenced on the Illumina MiSeq platform. Sequences were clustered into operational taxonomic units (OTUs) using the Uparse algorithm (v7.0.1001). The beta diversity of the microbiota and linear discriminant analysis (LDA) was performed using the Qiime and LDA effect size analysis (LEfSe) softwares, respectively. Relative abundance and statistical difference analyses of microbiota at the phylum, family, and genus levels, as well as correlation analyses and heat mapping, were performed by R software.

### Statistical analysis

Statistical analysis was conducted using SPSS 26.0 software. Multiple group comparisons were evaluated using one-way analysis of variance (ANOVA) and Tukey’s post hoc test, and the linear and quadratic effects were investigated using orthogonal polynomial regression. Two-group comparisons were evaluated using Student’s *t*-test. The results are presented as mean ± SD. The results were visualized by GraphPad Prism software (version 8.0).

## Results

### CGA supplementation improved egg performance and quality in post-peaking laying hens

As shown in Table 2, CGA400 significantly increased the LR compared to CON during weeks 6–10 and weeks 1–10 (*P* < 0.05). During these two periods, there were linear and quadratic increases in the LR with CGA supplementation (*P* < 0.05). At weeks 1–5, weeks 6–10 and weeks 1–10, CGA400 significantly increased the ADEM compared to CON (*P* < 0.05). CGA200 and CGA800 significantly increased the ADEM compared to CON during weeks 6–10 and weeks 1–10 (*P* < 0.05). However, throughout the experiment, CGA supplementation showed no significant effect on the ADFI and FCR of post-peaking laying hens (*P* > 0.05).

**Table 2:** Effects of CGA on laying performance of post-peaking laying hens

**Items**	**Dietary CGA level, mg/kg**				***P*-value**		
	**0 (CON)**	**200**	**400**	**800**	**ANOVA**	**Linear**	**Quadratic**
LR, %							
Weeks 1−5	85.61 ± 3.57	88.03 ± 3.32	88.79 ± 2.75	88.10 ± 3.68	0.401	0.196	0.269
Weeks 6 −10	77.18 ± 4.62^b^	83.81 ± 5.87^ab^	86.45 ± 2.89^a^	83.11 ± 3.19^ab^	0.010	0.017	0.010
Weeks 1−10	81.40 ± 3.89^b^	86.14 ± 4.08^ab^	87.62 ± 2.73^a^	85.60 ± 2.84^ab^	0.031	0.036	0.026
ADEM, g/hen/d							
Weeks 1−5	51.05 ± 1.62^b^	53.19 ± 1.59^ab^	54.62 ± 1.28^a^	53.08 ± 1.13^ab^	0.003	0.009	0.005
Weeks 6−10	49.55 ± 1.36^b^	52.94 ± 1.32^a^	53.38 ± 1.25^a^	52.32 ± 0.84^a^	< 0.001	0.001	< 0.001
Weeks 1−10	50.30 ± 1.44^b^	53.07 ± 1.70^a^	54.00 ± 1.61^a^	52.70 ± 1.50^a^	0.004	0.010	0.005
ADFI, g/hen/d							
Weeks 1−5	112.50 ± 4.73	115.36 ± 2.03	115.87 ± 1.29	114.10 ± 2.10	0.068	0.032	0.105
Weeks 6−10	111.13 ± 3.12	114.05 ± 2.91	115.71 ± 3.18	114.63 ± 2.20	0.208	0.321	0.061
Weeks 1−10	111.82 ± 3.91	114.71 ± 2.31	115.79 ± 2.02	114.37 ± 1.97	0.101	0.089	0.062
FCR, g/g							
Weeks 1−5	2.21 ± 0.10	2.17 ± 0.05	2.12 ± 0.06	2.15 ± 0.06	0.230	0.114	0.254
Weeks 6−10	2.25 ± 0.10	2.16 ± 0.05	2.17 ± 0.10	2.19 ± 0.05	0.269	0.355	0.101
Weeks 1−10	2.22 ± 0.08	2.16 ± 0.07	2.15 ± 0.08	2.17 ± 0.06	0.352	0.227	0.183

^a–c^Values with different superscripts in a row indicate significant differences (*P* < 0.05). All values are expressed as means of 6 replicates per treatment

*LR* Laying rate, *ADEM* Average daily egg mass, *ADFI* Average daily feed intake, *FCR* Feed conversion ratio

Eggshell weight, strength, and thickness were significantly increased (*P* < 0.05) in CGA400 group than those in CON group (Table 3). Similarly, compared to the CON group, eggshell weight and eggshell strength were significantly enhanced in CGA800 group (*P* < 0.05). The CGA200 group showed enhanced eggshell-related indicators in varying degrees, although no statistically significant differences were observed (*P* > 0.05). No significant differences showed in the albumen height or Haugh units among groups (*P* > 0.05).

**Table 3:** Effects of CGA on egg quality of post-peaking laying hens

Items	Dietary CGA level, mg/kg				*P*-value		
	**0 (CON)**	**200**	**400**	**800**	**ANOVA**	**Linear**	**Quadratic**
Egg shape index	1.31 ± 0.05	1.31 ± 0.03	1.32 ± 0.04	1.34 ± 0.04	0.643	0.304	0.481
Eggshell weight, g	8.20 ± 0.24^b^	8.41 ± 0.28^ab^	8.92 ± 0.55^a^	8.77 ± 0.44^a^	0.021	0.113	0.289
Eggshell ratio, %	13.25 ± 1.01	13.44 ± 0.47	14.08 ± 0.46	13.87 ± 0.69	0.180	0.352	0.489
Eggshell strength, kgf/cm²	2.89 ± 0.40^b^	3.35 ± 0.75^ab^	3.84 ± 0.54^a^	3.81 ± 0.41^a^	0.020	0.183	0.005
Eggshell thickness, mm	0.31 ± 0.02^b^	0.33 ± 0.01^ab^	0.34 ± 0.01^a^	0.34 ± 0.02^ab^	0.038	0.010	0.235
Yolk weight, g	16.82 ± 0.86	17.81 ± 1.69	17.51 ± 1.01	17.96 ± 1.17	0.401	0.111	0.588
Egg yolk ratio, %	27.00 ± 1.41	28.33 ± 2.07	27.83 ± 0.98	28.50 ± 1.76	0.391	0.104	0.617
Yolk color	5.33 ± 0.82	5.50 ± 0.84	5.67 ± 0.52	6.00 ± 0.00	0.339	0.132	0.753
Albumen height, mm	7.07 ± 0.84	7.28 ± 0.32	8.10 ± 1.40	7.18 ± 0.85	0.241	0.501	0.154
Haugh unit	81.44 ± 4.96	84.50 ± 2.38	84.80 ± 1.53	84.93 ± 3.15	0.229	0.085	0.283

^a–c^Values with different superscripts in a row indicate significant differences (*P* < 0.05). All values are expressed as means of 6 replicates per treatment

### CGA supplementation enhanced ovarian structure and function in post-peaking laying hens

**Fig. 1:**
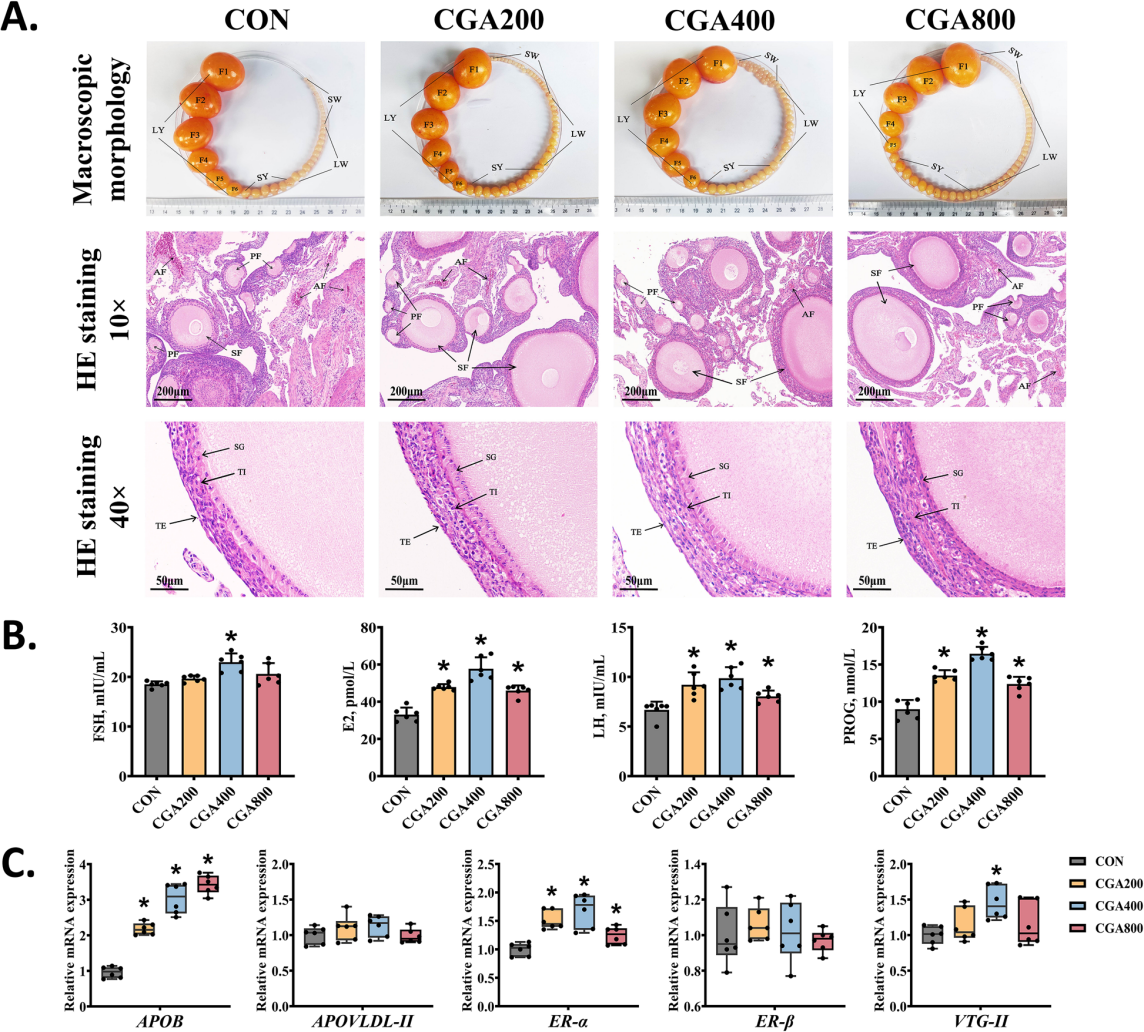
CGA improved follicular morphology and promoted reproductive hormone levels and yolk precursor synthesis in post-peaking laying hens. **A** Macromorphological photographs of follicles and representative histological images of the ovarian stroma. **B** Levels of serum reproductive hormones (*n* = 6). **C** Relative mRNA expression levels of genes associated with yolk precursor synthesis in liver detected by qPCR (*n* = 6). All data were represented as mean ± SD. *P < 0.05 compared to the CON group

Compared to the CON group, CGA inhibited the reduction of total follicle number, with a more pronounced effects on preovulatory follicles SY and SW (Fig. 1A). Regarding the microstructure of follicles, CGA resulted in a reduction in follicle atresia and an improvement in both follicle size and membrane thickness, whereas the CON hens had a lower number of follicles, smaller follicular morphology, and thinner follicular membranes (black arrows). Compared to the CON group, CGA supplementation, regardless of the dose, increased serum LH, E2, and PROG levels (*P* < 0.05; Fig. 1B). In addition, serum FSH levels increased in the CGA400 group as compared to CON (*P* < 0.05).

To further investigate the molecular mechanisms by which CGA improved production performance, the mRNA expression of yolk precursor-related genes associated with follicular energy uptake and developmental maturation was determined. As depicted in Fig. 1C, CGA significantly increased (*P* < 0.05) the mRNA expression levels of *APOB* and *ER-α* and 400 mg/kg of CGA supplementation also significantly increased *VTG-II* mRNA expression in liver (*P* < 0.05) as compared to the CON group. However, there was no statistical difference in mRNA expression levels of *APOVLDL-II* and *ER-β* in liver among groups (*P* > 0.05).

### CGA alleviated the hepatic inflammatory responses and oxidative stress in post-peaking laying hens

**Fig. 2:**
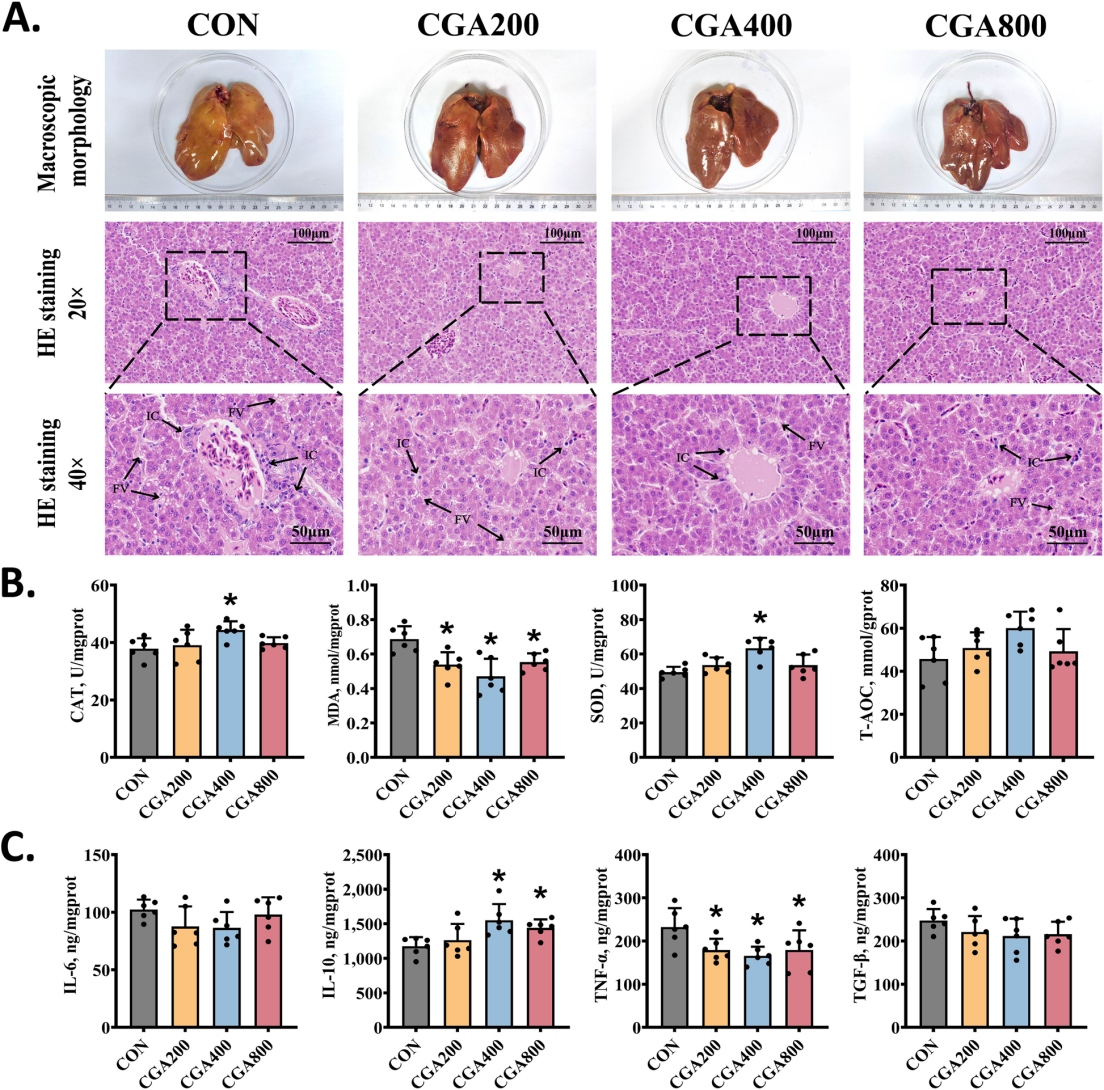
CGA ameliorated hepatic oxidative stress and inflammatory response in post-peaking laying hens. **A** Macromorphological photographs and representative histological images of the liver. **B** Levels of liver oxidative stress parameters (*n* = 6). **C** Levels of liver inflammatory associated parameters (*n* = 6). All data were represented as mean ± SD. *P < 0.05 compared to the CON group

Macroscopic and microscopic images of the liver were shown in Fig. 2A. Compared to the CON hens, the livers of the CGA-treated hens had less lipid vacuole accumulation and inflammatory cell infiltration. CGA significantly reduced the lipid oxidation end product, MDA, in the liver of post-peaking hens (*P* < 0.05; Fig. 2B). The activities of CAT and SOD and the mRNA levels of antioxidant-related genes (e.g., *HO-1*, *PRDX-3* and *GCLM*) were significantly upregulated (*P* < 0.05) in hens from the CGA400 group (Fig. 2B and Fig. S1A). CGA significantly regulated the mRNA expression levels of pro- and anti-inflammatory genes (*IL-1β*, *IL-4, IL-6*, and *TNF-α*) (Fig. S1B) and demonstrated a significant decrease in TNF-α levels (Fig. 2C). Additionally, both CGA400 and CGA800 groups exhibited a significant increase in IL-10 levels in liver (*P* < 0.05).

### CGA-induced changes in the liver transcriptome in post-peaking laying hens

**Fig. 3:**
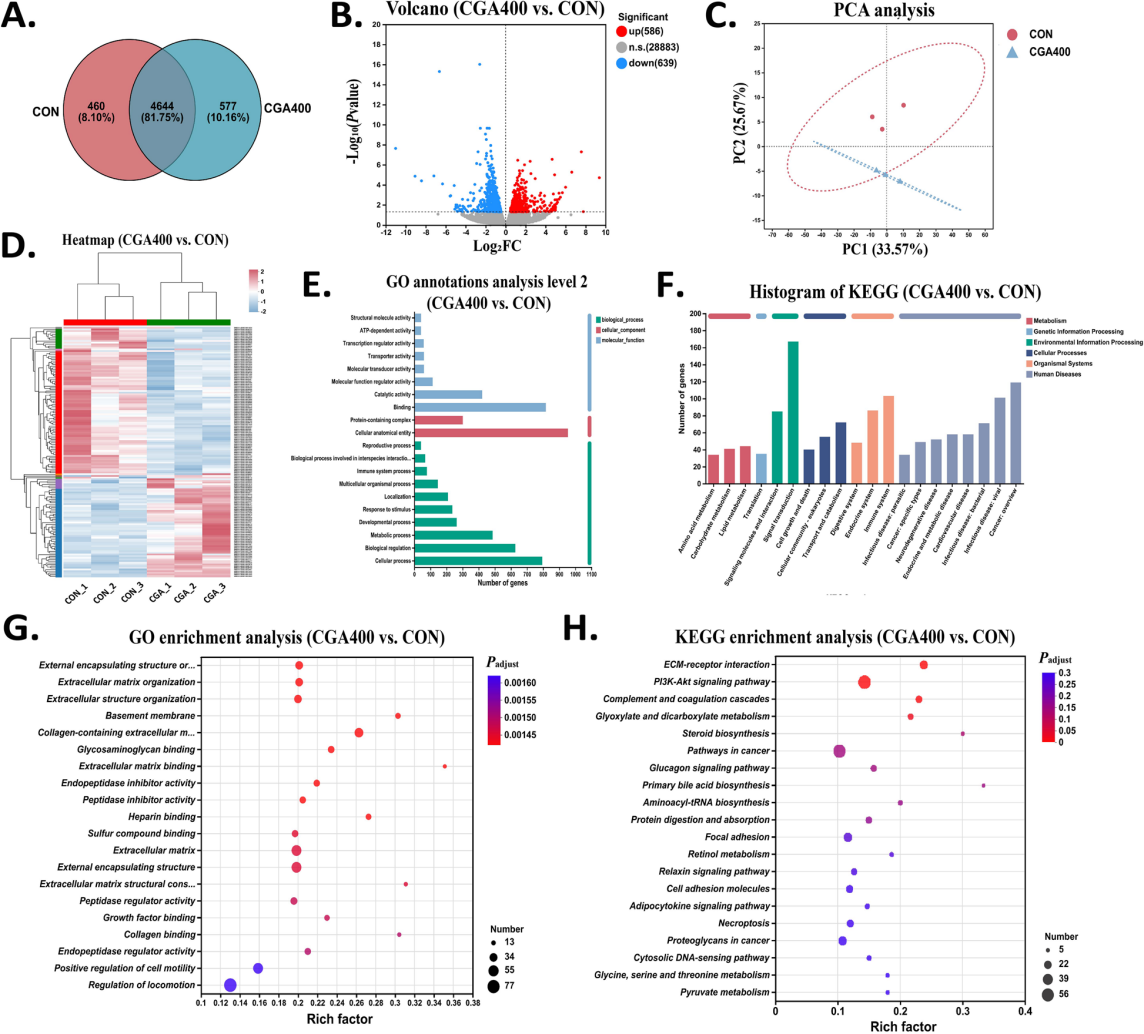
Transcriptome reveals the mechanisms underlying the chlorogenic acid-mediated attenuation of oxidative stress and systemic inflammatory responses (*n* = 3). **A** Venn diagram for visualising of mutual and unique genes. **B** Volcano plot of differentially expressed genes (DEGs). **C** Principal component analysis (PCA) score plot. **D** Cluster analysis heatmap of gene expression patterns in gene sets. **E** and **F** Histogram of GO and KEGG enrichment analysis of DEGs. **G** and **H** Scatter plot of GO and KEGG enrichment analysis of DEGs

Based on the phenotype results, liver RNA sequencing was performed for both the CON and CGA400 groups. The Fig. 3A presented the VENN diagram comparing the two groups. Specifically, 4,644 genes were shared between the two groups, with 460 and 577 genes unique to the CON and CGA400 groups, respectively. The volcano plot in Fig. 3B showed the upregulation and downregulation of DEGs between the CON and CGA400 groups. A total of 1,225 DEGs were identified, of which 586 were upregulated and 639 were downregulated. The PCA score plot and cluster analysis heat map demonstrated clear differences in clustering between the CON and CGA400 groups based on gene expression levels (Fig. 3C and D). According to the GO annotation bar plot, DEGs between the CON and CGA400 groups were primarily enriched in the regulation of reproductive, immune, and metabolic processes (Fig. 3E). Concurrently, the KEGG annotation bar plot showed that DEGs were enriched in the regulation of carbohydrate and lipid metabolism, cell growth and death, and the immune system (Fig. 3F). DEGs between the CON and CGA400 groups were also involved in GO terms such as the organization of external encapsulating structure, extracellular structure, and basement membrane (Fig. 3G). Additionally, the PI3K/AKT signaling pathway, steroid biosynthesis, primary bile acid, and adipocytokine signaling pathway were enriched in KEGG enrichment analysis (Fig. 3H). These findings suggested that CGA may be involved in specific biological processes by regulating DEGs expression, thereby improving liver health in post-peaking laying hens.

### CGA promotes hepatic lipid metabolism by regulating adipocytokine signaling pathway-related genes and proteins

**Fig. 4:**
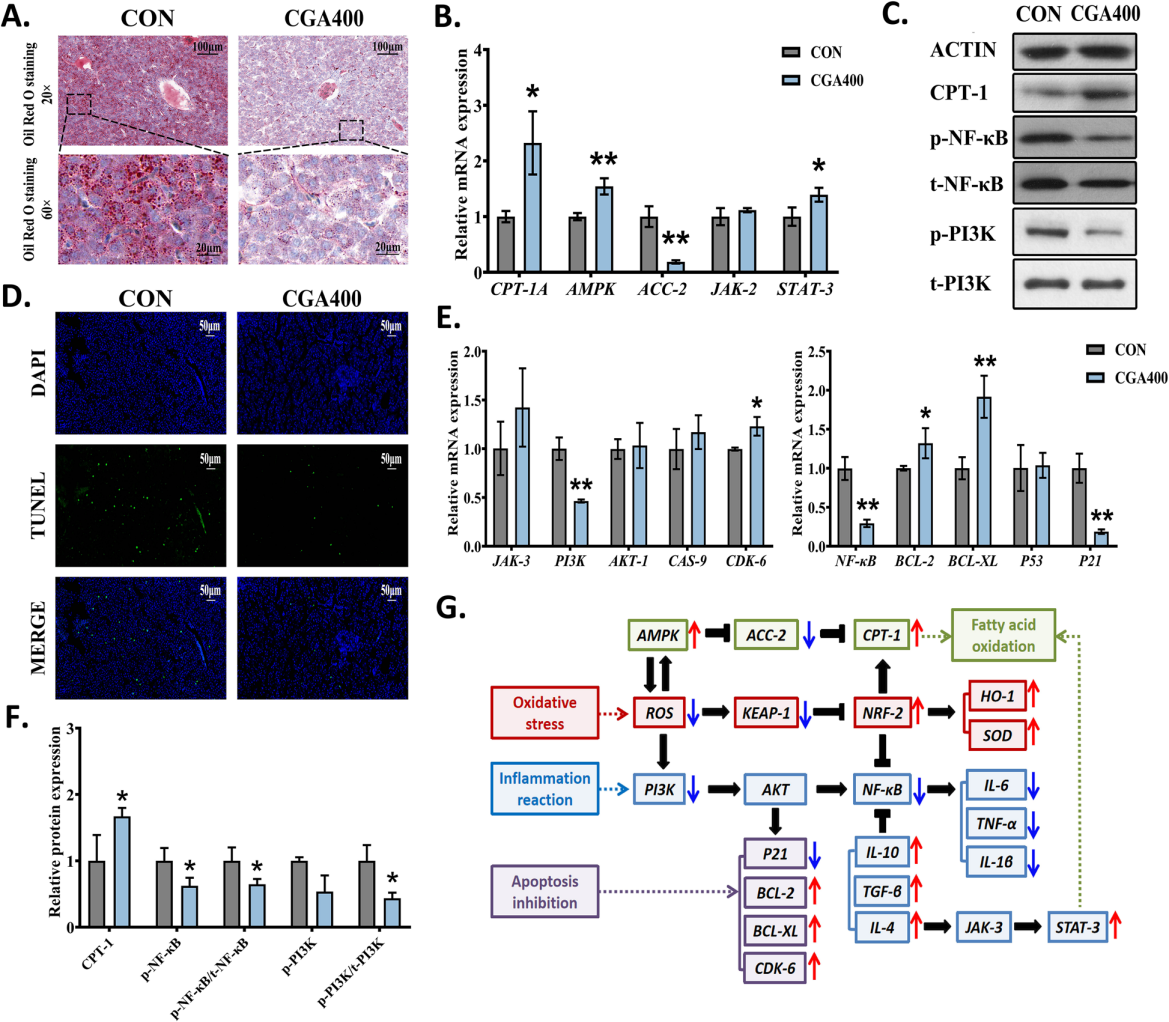
Revalidation of the expression of signaling pathway genes screened in the KEGG enrichment analysis results. **A** Representative Oil Red O staining images of liver. **B** Relative mRNA expression levels of DEGs associated with adipocytokine signaling pathway detected by qPCR (*n* = 6). **C** The representative western blot images. **D** Representative TUNEL staining images of liver. **E** Relative mRNA expression levels of DEGs associated with the PI3K-AKT signaling pathway and its downstream genes expression detected by qPCR (*n* = 6). **F** Relative expression levels of adipocytokine signaling pathway-related protein (CPT-1A) and PI3K-AKT signaling pathway-related proteins (p-PI3K/t-PI3K and p-NF-κB/t-NF-κB) in CON and CGA400 groups (*n* = 3). **G** Schematic diagram of the effect of CGA on regulatory cascades in the liver. All data were represented as mean ± SD. *P < 0.05 and **P < 0.01 compared with the CON group

Based on the GO and KEGG annotation results, we initially focused on the adipocytokine signaling pathway associated with lipid metabolism. As shown in Fig. 4A, Oil Red O staining revealed the dense red lipid droplets in the livers of the CON group, indicating lipid accumulation. However, after CGA400 supplementation, the lipid droplets were notably reduced and smaller. This modulatory effect corresponded to the reduction in fat vacuoles by CGA in the HE staining images (Fig. 2A), again demonstrating significant positive effects of CGA on hepatic lipid regulatory processes.

To determine the potential molecular mechanisms through which CGA improved lipid metabolism, the expression of DEGs in the adipocytokine pathway enriched in KEGG was validated using qPCR. CGA400 significantly increased the gene expression of the fatty acid β-oxidation rate-limiting enzyme *CPT-1A* (*P* < 0.05; Fig. 4B). The mRNA levels of *AMPK* and *STAT-3* (upstream genes of *CPT-1A*) were significantly elevated (*P* < 0.05), whereas the level of *ACC-2* was significantly downregulated (*P* < 0.05). In addition, CGA400 significantly increased the protein expression level of CPT-1A (*P* < 0.05; Fig. 4C and F).

### CGA ameliorated hepatic apoptosis and inflammation by regulating the PI3K/AKT signaling pathway-related genes and proteins

The TUNEL images in Fig. 4D showed a weakened green fluorescence signal in liver cells of the CGA400 group compared to the CON group, indicating a reduction in apoptotic cells. Based on the KEGG enrichment results, the levels of PI3K/AKT pathway-related hepatic inflammation and apoptosis genes were further analyzed. As shown in Fig. 4E, CGA400 significantly downregulated the mRNA expression level of the *PI3K* (*P* < 0.05). The mRNA expression levels of *JAK-3* (an upstream gene of *PI3K*) and *AKT* (downstream gene of *PI3K*) tended to increase; however, there were no statistically significant differences. CGA400 downregulated the mRNA levels of inflammation-related genes *NF-κB* (*P* < 0.05), downregulated the mRNA levels of apoptosis-related genes *P21* (*P* < 0.05), and upregulated the mRNA levels of apoptosis-related genes *CDK-6*, *BCL-2* and *BCL-XL* (*P* < 0.05). In addition, CGA400 significantly decreased the protein expression levels of p-PI3K/t-PI3K, p-NF-κB and p-NF-κB/t-NF-κB (*P* < 0.05; Fig. 4C and F). The simplified diagram of the regulatory cascade effect of CGA was shown in Fig. 4G.

### CGA ameliorated intestinal oxidative stress and inflammation in post-peaking laying hens

**Fig. 5:**
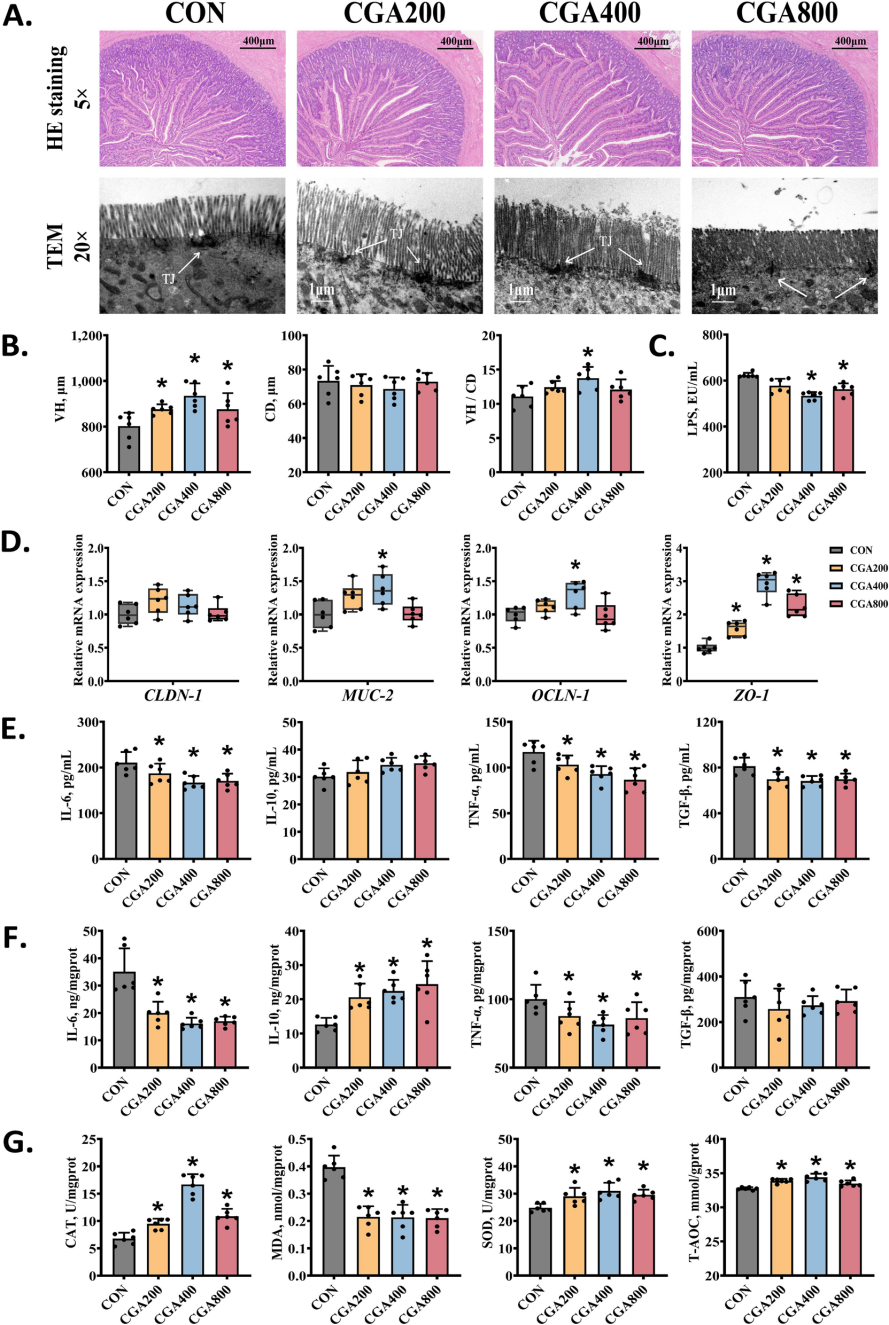
CGA improved intestinal morphology and barrier function of the jejunum in post-peaking laying hens. **A** and **B** Representative histological images and morphological indicators of the jejunum. **C** Levels of serum LPS (*n* = 6). **D** Relative mRNA expression levels of genes associated with tight junction in jejunum detected by qPCR (*n* = 6). **E** Levels of serum inflammatory associated parameters (*n* = 6). **F** Levels of jejunum inflammatory associated parameters (*n* = 6). **G** Levels of jejunum oxidative stress associated parameters (*n* = 6). All data were represented as mean ± SD. *P < 0.05 compared with the CON group

The microstructures of the jejunum of the CON and CGA groups were shown in Fig. 5A, the intestinal villi of each group were neatly arranged. The histograms in Fig. 5B showed that intestinal villus length was significantly increased in both the CGA200 and CGA400 groups, and crypt depth was also significantly decreased in the CGA400 group as compared to the CON group (*P* < 0.05). Additionally, the TEM images showed that hens in the CGA groups had longer and denser microvilli, as well as more structurally defined and intact tight junctions than those in the CON group (Fig. 5A), which was verified by the gene levels of tight junction proteins and mucin. As shown in Fig. 5D, CGA supplementation significantly increased the mRNA levels of *MUC-2*, *OCLN-1*, and *ZO-1* (*P* < 0.05), while CGA had no significant effect on *CLDN-1*.

Compared to the CON group, CGA reduced the levels of serum LPS, IL-6, TNF-α and TGF-β in different degrees, with CGA400 having the most pronounced effect (Fig. 5C and E). The Fig. 5F indicated that CGA groups significantly downgraded the jejunal levels of IL-6, TNF-α and TGF-β, and significantly increased the level of IL-10. Notably, the CGA400 and CGA800 groups significantly downregulated the mRNA expression of *IL-6*, *TNF-α* and *TGF-β*, and significantly upregulated the mRNA expression of *IL-4* and *IL-10* (Fig. S1D), consistent with the results in Fig. 5F. In addition, CGA significantly increased the activities of CAT, SOD, and T-AOC and significantly decreased the level of MDA (*P* < 0.05; Fig. 5G). CGA significantly regulated *KEAP-1* and *NRF-2* expression in the jejunum, and CGA400 upregulated the expression of the antioxidant enzyme genes *HO-1*, *GST*, *SOD-1*, *SOD-2*, *PRDX-3*, and *GCLM* (*P* < 0.05; Fig. S1C).

### CGA supplementation improved gut microbiota in post-peaking laying hens

**Fig. 6:**
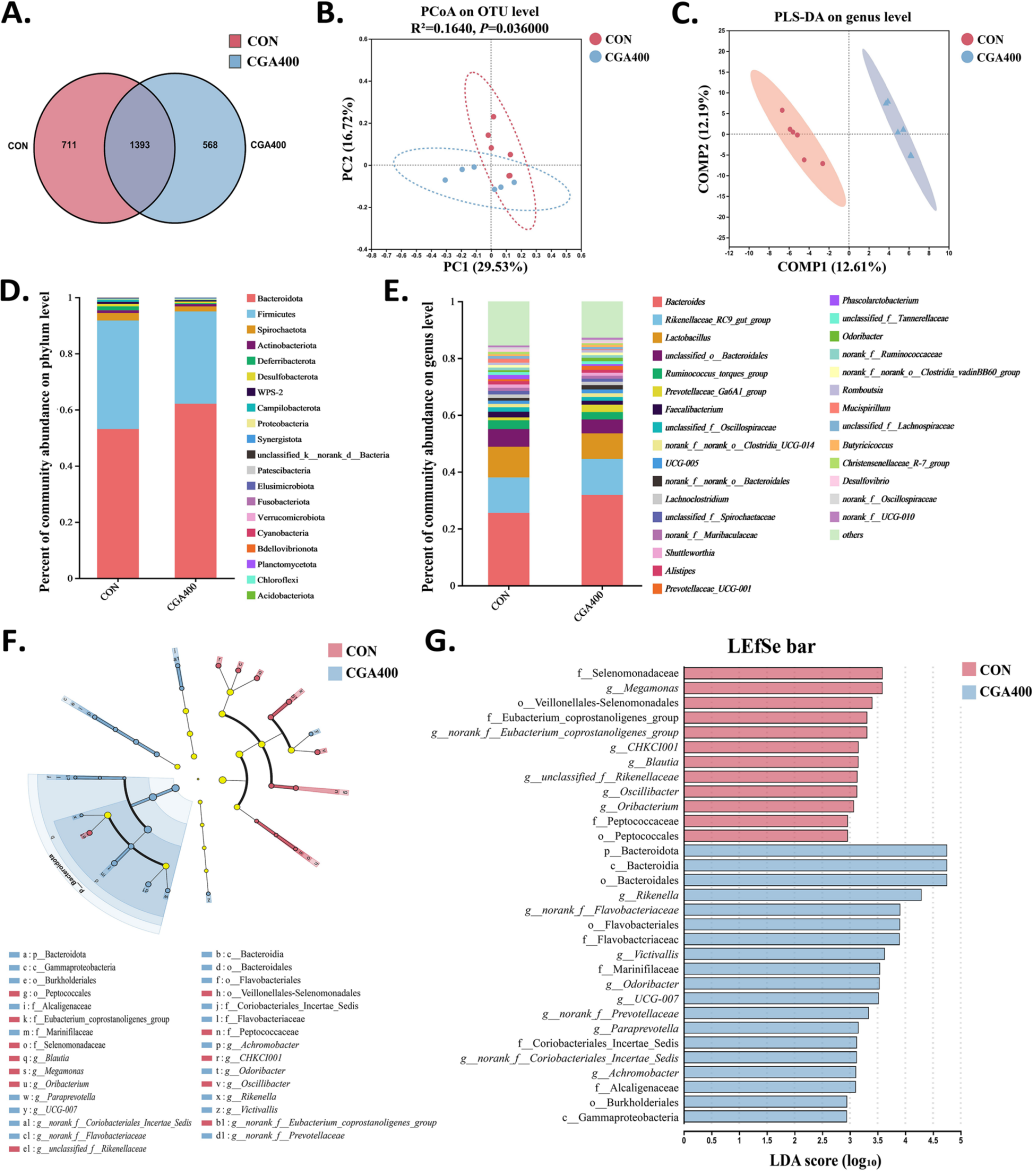
CGA regulated the diversity and composition of cecal microbiota of post-peaking laying hens (*n* = 6). **A** Venn plot of visualizing mutual and unique OTUs. **B** Principal coordinate analysis (PCoA) score plot at OTU level. PC1, the first principal component. PC2, the second principal component. **C** Partial least squares discriminant analysis (PLS-DA) analysis score plot at genus level. COMP1, the first component. COMP2, the second component. **D** and **E** Microbiota composition barplot on phylum and genus levels. **F** Cladogram of LEfSe analysis. **G** Histogram of LEfSe analysis

**Fig. 7:**
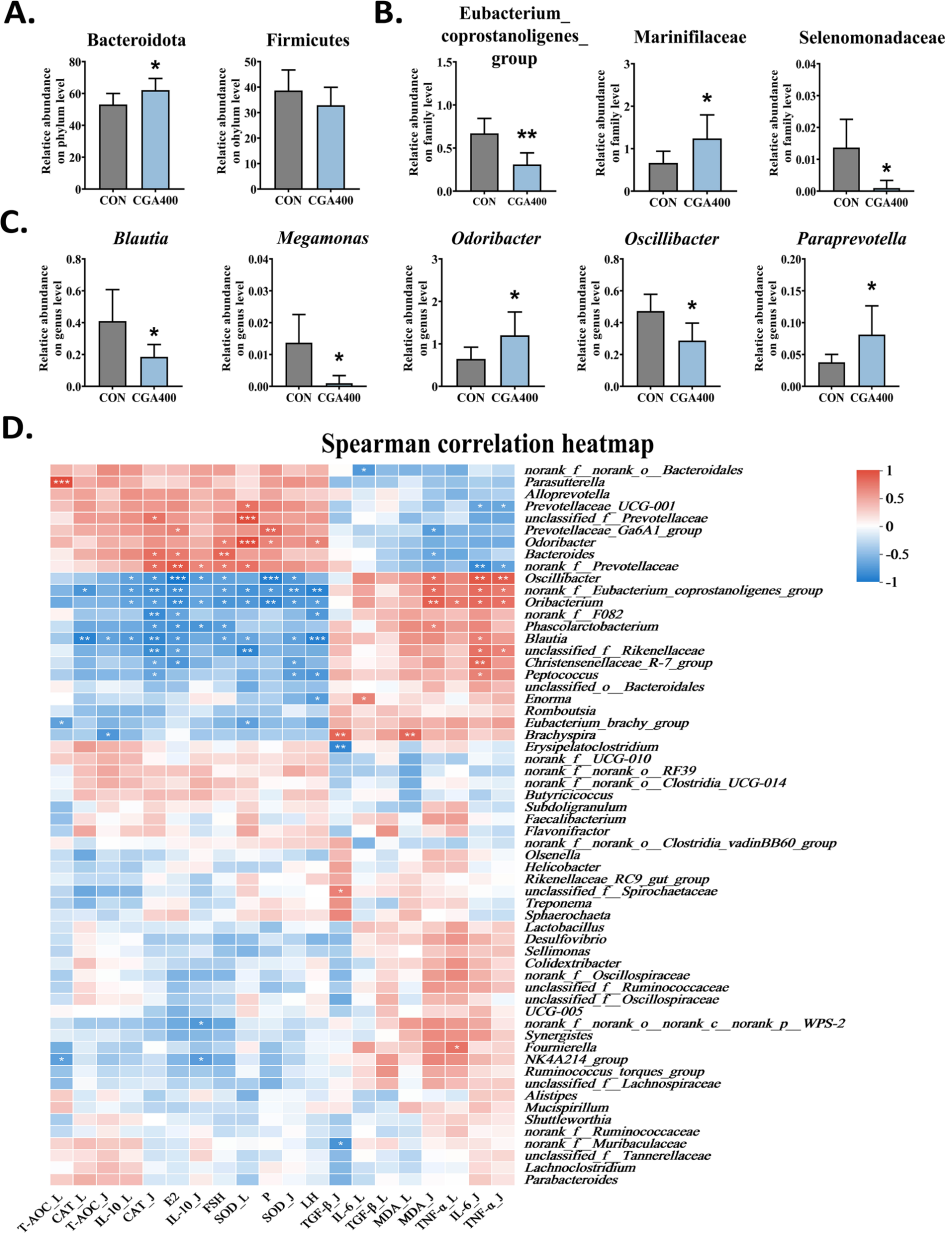
CGA regulated the specific microbiota in cecal contents and alleviated oxidative stress and inflammatory response along gut-liver axis. **A–C** Relative proportion of microbiota on phylum (**A**), family (**B**), and genus (**C**) levels. All data were represented as mean ± SD. *P < 0.05 and **P < 0.01 compared to the CON group. **D** Correlation analysis of cecal microbiota with indicators related to reproductive performance and parameters related to oxidative stress. _L, indicators of liver, _J, indicators of jejunum

The 16S rDNA sequencing results for both the CON and CGA400 groups were shown in Fig. 6. The VENN diagram showed that 1,393 OTUs were shared between the two groups, with 711 OTUs unique to the CON group and 568 OTUs unique to the CGA400 group (Fig. 6A). The PCoA score plot depicted in Fig. 6B indicates a significance (*P* < 0.05) between the microbiota of the CON and CGA400 groups, with PC1 and PC2 contributing 29.53% and 16.72% to the sample difference, respectively. Similarly, the PLS-DA model revealed significant differences between the microbiota in the CON and CGA400 groups (Fig. 6C). As demonstrated in Fig. 6D and E, CGA affected the relative abundance of the gut microbiota at both the phylum and genus levels. At the phylum level, the microbiota with high relative abundance were Bacteroidota, Firmicutes, Spirochaetota, and Actinobacteriota. At the genus level, the microbiota with high relative abundance were *Bacteroides*, *Rikenellaceae_RC9_gut_group*, *Lactobacillus*, and *Ruminococcus_torques_group*. Based on the LDA and LEfSe results, we identified signature microbial taxa that were significantly different between the CON and CGA400 groups (Fig. 6F and G). At the phylum level, CGA increased the relative abundance of Bacteroidota (*P* < 0.05), and decreased that of Firmicutes (Fig. 7A). At the family level, Marinifilaceae was significantly increased in the CGA group, whereas Eubacterium_coprostanoligenes_group and Selenomonadaceae were significantly decreased (*P* < 0.05; Fig. 7B). At the genus level, *Odoribacter* and *Paraprevotella* were also significantly increased in CGA400 group, whereas *Blautia*, *Megamonas* and *Oscillibacter* were decreased as compared to the CON group (*P* < 0.05; Fig. 7C).

### Correlation analysis

To gain deeper insights into the serum hormone levels (FSH, LH, E2, and PROG), oxidative stress indicators (CAT, SOD, T-AOC and MDA), and inflammatory indicators (IL-6, IL-10, TNF-α and TGF-β) content and the abundance of cecal microbiota, the Spearman correlation was performed and the correlation heatmap was displayed in Fig. 7D. Antioxidant indicators (CAT, SOD, and T-AOC), anti-inflammatory factors (IL-10), and reproductive hormones were similar in correlation analysis clustering. MDA and pro-inflammatory factors (IL-6, TNF-α and TGF-β), which were harmful to the organism, had similar patterns in correlation analysis clustering.

The levels of reproductive hormones, anti-inflammatory factors, and antioxidative factors were positively correlated with the relative abundance of *Bacteroides* and *Odoribacter* and negatively correlated with the relative abundance of *Blautia* and *Oscillibacter*. However, the correlation between the abundance of microbiota and levels of lipid peroxidation, MDA, and inflammatory factors was essentially the opposite. These findings suggested that the cecal microbiota may participated in CGA improvement of the gut-liver health of post-peaking hens.

## Discussion

In the context of intensive farming, the performance declination and egg quality deterioration of late-stage laying hens, as well as the development of aging-induced fat deposition, oxidative stress, and organ damage lesions, have presented significant challenges for the layer breeding industry [9, 23, 24]. CGA is an organic acid that exhibits prominent antibacterial, anti-inflammatory, antioxidant, and hepatoprotective activities in humans and rodents [13, 25]. In the present study, we found that the addition of a medium dose of CGA (400 mg/kg) significantly increased the LR, ADEM and improved egg quality indicators related to breakage resistance in laying hens. Contrastively, the low dose (200 mg/kg) and high dose (800 mg/kg) of CGA showed inferior improvement to the medium dose or were not significantly different from the CON group. This may be due to the fact that the low dose of CGA do not reach the physiological threshold for more significant effects in laying hens, whereas the high dose of CGA impose a potential burden on absorption and utilization in laying hens. However, the specific mechanisms of action of CGA in laying hens remain unclear.

The performance of laying hens is related to the quantity and quality of ovarian follicles, endocrine regulation, and yolk synthesis. During the reproductive process, the ovary forms several follicles, including preovulatory and pre-hierarchical follicles, which develop and mature to the discharge of eggs [26]. Follicular atresia is a physiological phenomenon in poultry. Studies have shown that an increase in the number of atretic follicles is correlated with decreased egg production, premature ovarian failure, and polycystic ovary syndrome [27, 28]. Therefore, promoting follicular development and reducing follicular atresia are conducive for improving the performance of laying hens. Serum reproductive hormone levels indicate reproduction in laying hens. FSH and LH stimulate follicle selection, granulosa cell differentiation, and steroid hormone synthesis [29–31]. Granulosa cells that undergo follicle selection synthesize PROG and E2 [32]. In this study, the CGA400 group showed a more significant improvement in ovarian health in laying hens compared to the CGA200 and CGA800 groups. Supplementation with 400 mg/kg of CGA inhibited the decrease in the number of pre-hierarchical and preovulatory follicles in post-peaking laying hens, improved the morphology of growing follicles SF and PF, reduced follicular atresia, and increased serum hormone levels. The liver-blood-ovary axis-mediated synthesis of yolk precursors revealed inseparable connection between liver and ovary. E2, produced by ovarian granulosa cells and entering the bloodstream, stimulates estrogen receptors (ER-α/β) in the liver, promoting the synthesis of yolk precursors such as vitellogenin (VTG-II), apolipoprotein B (APOB), and very-low-density apolipoprotein II (APOVLDLII), which are transported to the ovary to provide energy for follicular development [33, 34]. The results demonstrated that CGA400 significantly upregulated hepatic *APOB*, *ER-α*, and *VTG-II* expression, while having no significant effects on *ER-β*. This may be attributed to the fact that CGA significantly affected the E2 levels, as E2 stimulates the synthesis of yolk precursors mainly by stimulating *ER-α* rather than *ER-β* [33].

Aging in laying hens is closely related to events comprising cytoarchitectural lesions, oxidative stress, inflammatory damage, and lipid disorders in the liver [35]. Oxidative stress is a state of imbalance between the reactive oxygen radicals generated by organism and the antioxidant system to eliminate reactive oxygen species (ROS), which are closely related to the body’s antioxidant defenses, chronic inflammation, and lipid synthesis and accumulation [36, 37]. When the endogenous antioxidant system of the body declines insufficiently to resist ROS overproduction, oxidative stress is inevitable. In the present study, the livers of post-peaking laying hens in the CON group exhibited enlargement, greasy surfaces, an increased number of intercellular fat vacuoles, and inflammatory infiltrates. This suggested that the livers of late-stage laying hens without dietary intervention exhibited a certain degree of lipid degeneration and inflammatory damage. However, CGA treatment ameliorated these negative effects, especially in the CGA400 group. Several ex vivo and in vivo studies have validated the anti-inflammatory and antioxidant properties of CGA [38, 39]. Similarly, our results demonstrated the effectiveness of CGA as an exogenous antioxidant to alleviate oxidative stress and inflammation in laying hens. Dietary 400 mg/kg of CGA supplementation significantly increased the activity of antioxidant enzymes and contents of anti-inflammatory factors IL-10, while decreasing the levels of lipid peroxides MDA and TNF-α. Furthermore, CGA exerted a significant regulatory effect on a series of oxidative stress- and inflammation-related genes. NRF-2, an important transcription factor, is capable to regulate cell differentiation, proliferation and inflammation [40]. The cellular level of NRF-2 is maintained by KEAP-1 in basal conditions; however, under stressed conditions, NRF-2 translocates to nucleus and bind to antioxidant response element (ARE), thereby promoting the transcription of antioxidant enzymes [41]. Interestingly, we found that the KEAP-1/NRF-2/ARE pathway was regulated by CGA in the present study. By performing liver RNA sequencing of CON and CGA400 groups, we further observed that CGA-regulated DEGs were enriched in biological processes such as reproduction, immune system, and lipid metabolism. Additionally, the adipocytokine and PI3K-AKT signaling pathways were markedly enriched in the KEGG pathway analysis.

Fatty acid oxidation (FAO), the process by which lipoyl coenzyme A formed from fatty acid activation is transported to mitochondria and eventually oxidized to acetyl coenzyme A, is an important pathway for promoting fat consumption in tissues such as liver and muscle [42]. During FAO, lipoyl coenzyme A cannot pass directly into the inner mitochondrial membrane; however, CPT-1 converts it into lipoyl carnitine and transports it to the mitochondria [42]. CPT-1, the rate-limiting enzyme of FAO, is essential for the maintenance of lipid metabolism homeostasis, and its deficiency is associated with hepatic enlargement, liver failure, and muscle weakness [43, 44]. In the present study, CGA remarkably upregulated the mRNA expression level and the protein expression level of *CPT-1A*, a CPT-1 isoform predominantly expressed in the liver. In addition, a complex relationship exists between AMPK/ACC and FAO [45]. ACC catalyzes the carboxylation of acetyl coenzyme A to generate malonyl coenzyme A, which inhibits CPT-1 activity, thereby inhibiting FAO and reducing lipid metabolism [42]. AMPK phosphorylates and inactivates ACC [42]. Consequently, AMPK activation and ACC inactivation reduce hepatic lipogenic accumulation and steatosis [46]. Notably, the transcriptional levels of *AMPK* and *ACC-2* were significantly upregulated and downregulated, respectively, in the liver after CGA treatment in the present study, and the Oil Red O-stained images showed a significant amelioration in hepatic lipid accumulation after CGA treatment. Targeting JAK-2/STAT-3 has also been reported to be beneficial for the treatment of lipid metabolism disorders [47]. Activation of JAK-2 and phosphorylation of STAT-3 can promote CPT-1 expression, regulate body energy expenditure, and promote lipid metabolism [48]. In the present study, CGA treatment significantly upregulated *STAT-3* levels, but no significant changes were found in *JAK-2* levels. Our findings indicated that CGA ameliorated hepatic lipid deposition in laying hens by activating *CPT-1A* gene expression and protein expression, as well as regulating the expression of genes related to the AMPK/ACC-2 and JAK-2/STAT-3 pathways.

Obesity is a systemic state characterized by chronic low-level inflammation [49]. Excessive lipid accumulation leads to adipocyte hypertrophy, altering intracellular signaling, triggering intracellular stress responses, and activating inflammatory signaling pathways [50]. The PI3K/AKT pathway is a crucial signaling pathway involved in the regulation of inflammation, obesity, and immune diseases [51]. This pathway regulates PI3K and phosphatidylinositol (PIP3) production, which activates or inhibits downstream targets associated with cell proliferation, apoptosis, and inflammation by regulating the recruitment of the effector protein AKT [51]. In this study, CGA remarkably downregulated the mRNA expression and the protein expression of PI3K and NF-κB in liver, with a tendency to downregulate the mRNA expression of *AKT* but not significantly. PI3K is implicated in the developmental differentiation, activation, and migration of immune cells [51]. The activated PI3K stimulates AKT and its downstream NF-κB leading to the transcription of TNF-α and IL-1β, and is also involved in the process of IL-6 secretion by dendritic cells under the stimulation of CD80/CD86 [52, 53]. Conversely, the inhibition of PI3K suppresses pro-inflammatory factor secretion and promotes the secretion of IL-10 [54]. Our results are in partial agreement with these previous findings. Interestingly, we also found that the *P21* gene, downstream of *AKT*, was significantly reduced, whereas the gene transcript levels of *CDK-6*, *BCL-2*, and *BCL-XL* were significantly increased. CDK-6 is one of the cyclin-dependent protein kinases (CDKs) which active in cell cycle phases and cell proliferation [55]. As typical senescence-associated secretory phenotype factors, P21 and P53 are associated with the promotion of cell arrest and apoptosis, recruitment of immune cells, and exacerbation of the inflammatory milieu [56]. Activation of P53 and P21 signaling has been reported to inhibit hepatocyte proliferation and accelerate senescence [56, 57]. BCL-2 and BCL-XL are momentous members in the BCL-2 family that are involved in mitochondria-mediated apoptotic pathways and maintenance of cellular homeostasis [58]. Our results suggested that CGA reduced inflammation levels by inhibiting the mRNA and protein expression of PI3K and its downstream NF-κB, and promoted cellular homeostasis by regulating cell proliferation and apoptosis-related genes, which ultimately ameliorates liver injury in post-peaking laying hens. Overall, CGA may achieve multi-target interconnections and actions mainly through the regulation of KEAP-1/NRF-2/ARE, PI3K/AKT, and AMPK/ACC-2/CPT-1 pathways, thus improving lipid metabolism, oxidative stress, and inflammatory response in laying hens.

A growing body of research has conclusively shown that the gut and its microbiota interact with the liver through multiple mechanisms [59, 60]. Current research points to four categories of interactions in the gut-liver axis: interactions between the microbiota and intestinal barrier, interactions between the microbiota and liver cells, interactions between the microbiota and immune cells in the blood, and interactions between the microbiota in the gut [61]. Proper functioning of the gut-liver axis depends on an intact intestinal barrier. Intestinal barriers include mechanical, biological, immune, and chemical barriers, which together control the transport of substances in the gut [60]. However, when the intestinal barrier is dysfunctional or its permeability is increased, metabolites and pathogen-associated molecular patterns (PAMPs) from the gut microbiota are translocated, disrupting the homeostasis of the internal environment [62]. In the present study, CGA improved jejunal morphology, tight junction morphology, and tight junction gene expression. In addition, CGA400 significantly attenuated jejunal oxidative stress through upregulating the NRF-2/ARE pathway and increasing the expression of antioxidant enzyme genes. Notably, CGA400 significantly reduced the serum LPS level. LPS, a typical PAMP and a crucial indicator of intestinal permeability, can cross the intestinal epithelial barrier, trigger an immune response, and exacerbate the upregulation of pro-inflammatory factors in blood circulation and in the liver [63]. Therefore, increased intestinal permeability is likely to lead to increased chronic systemic inflammation. Systemic inflammation and PAMPs synergistically exacerbate the development of hepatic fibrosis, a universal symptom of chronic disease, which is associated with ROS, inflammatory factors, and hepatic stellate cells (HSCs) activation [64, 65]. TGF-β is a cytokine activated by aging and oxidative stress [66]. Oxidative stress-induced liver fibrosis was found to be associated with activation of the TGF-β/SMAD pathway, while inhibition of TGF-β signaling is one of the important targets for the treatment of numerous chronic liver diseases [65, 67]. These results demonstrated the ability of CGA to inhibit intestinal oxidative stress, barrier damage, and endotoxin translocation and to ameliorate the organic health of post-peaking laying hens by reducing systemic inflammation.

Gut microbiota has been shown to be strongly associated with chronic diseases comprising obesity, NAFLD, and cirrhosis [61]. As the two most dominant microorganisms in the cecal of laying hens, Firmicutes are associated with the promotion of obesity and increased levels of endotoxins and inflammation [68], whereas Bacteroidota have the opposite effects [69]. The elevated Firmicutes/Bacteroidota ratios were correlated with the development of obesity and cardiovascular disease [70, 71]. In this study, we found that CGA reduced the relative abundance of Firmicutes and significantly increased the relative abundance of *Bacteroidota* in laying hens. Changes in the relative abundance of *Eubacteria_coprostanoligenes_group* and *Blautia* have been reported to be strongly associated with obesity and inflammatory progression [72–74]. CGA significantly reduced the relative abundance of these two microorganisms, which is unanimous with a previous study [75]. Similarly, the abundance of systemic inflammation-associated microbiota such as *Selenomonadaceae* and *Megamonas* [76], was strikingly reduced in the CGA-treated group. *Marinifilaceae* was correlated with good glycemic control [77]. *Odoribacter*, a group of short-chain fatty acid-producing microorganisms whose abundance is negatively associated with the emergence of NAFLD, cystic fibrosis, and inflammatory bowel disease, are beneficial commensal bacteria that interact with the host [78]. The increased *Oscillibacter* was closely associated with intestinal permeability and inflammation [79]. In this study, CGA increased the relative abundance of *Marinifilaceae* and *Odoribacter* and decreased the relative abundance of *Oscillibacter*. Notably, CGA treatment also increased *Paraprevotella*, which can regulate intestinal trypsin activity and is beneficial for the prevention and treatment of bacterial and viral intestinal infections [80]. The microbiota also significantly correlated with reproductive hormones, oxidative stress, and inflammation levels in laying hens. Our results suggested that the modulation of gut microbiota structure and abundance by CGA played a beneficial role in promoting the health of the gut and liver in post-peaking laying hens.

## Conclusion

**Fig. 8:**
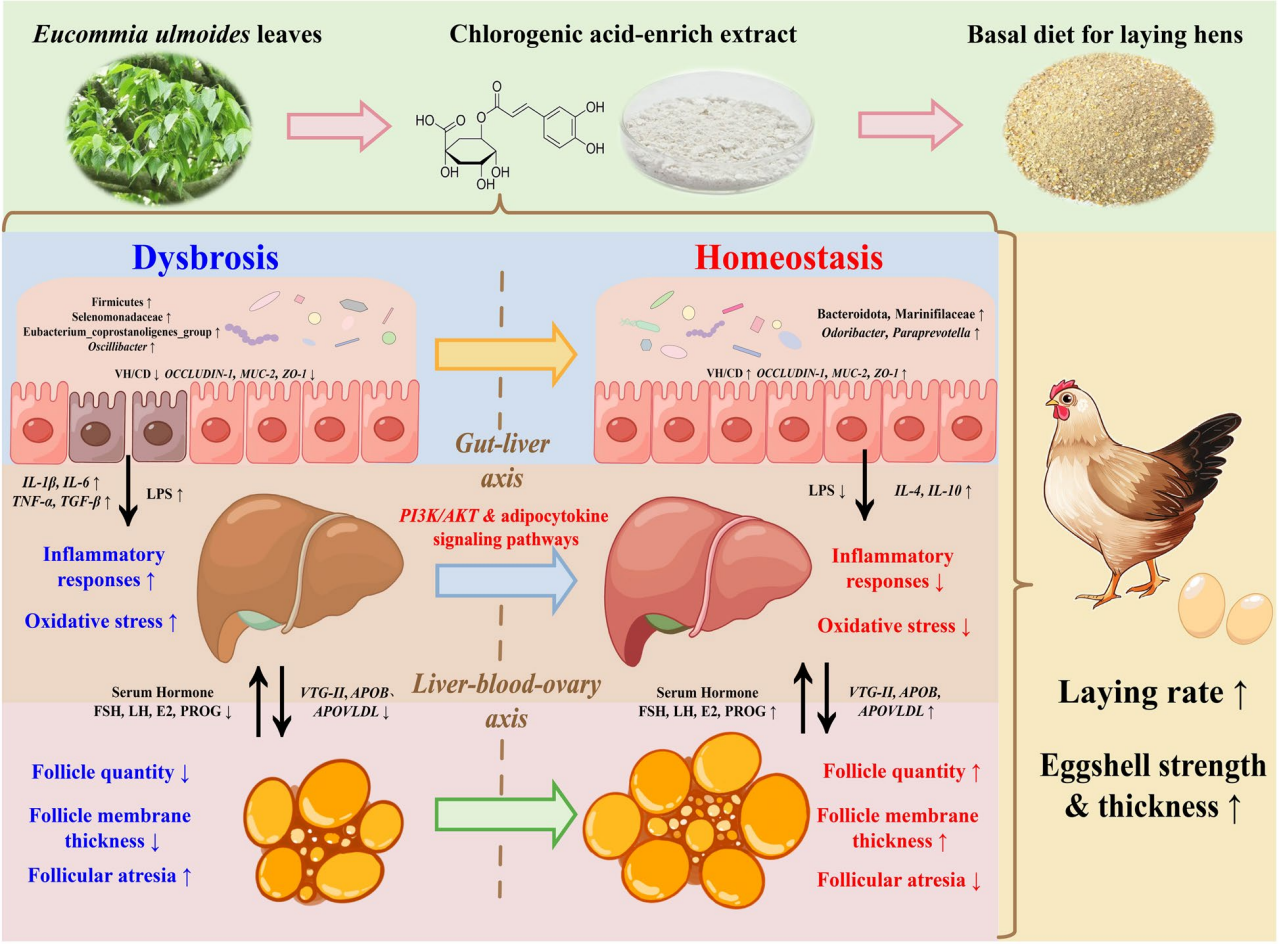
Schematic diagram of regulatory network of CGA-mediated attenuation of oxidative stress and systemic inflammatory responses

In conclusion, CGA alleviated multi-organ damage and dysfunction by suppressing the systemic inflammatory responses and oxidative stress in post-peaking laying hens, thereby improving egg performance and quality (Fig. 8). CGA could reinforce the intestinal barrier, diminish endotoxin transport, and regulate the structure of the cecal microbiota, thereby reducing systemic inflammation and maintaining the equilibrium of the internal environment within the organism. CGA improved liver lipid metabolism, inflammation, and apoptosis by modulating the adipocytokine and PI3K/AKT signaling pathways-related genes and protein. In addition, the regulatory mechanisms of CGA on gut and liver health in post-peaking laying hens may facilitate the recovery of ovarian function and promote reproductive hormone secretion and yolk precursor synthesis, thereby improving egg performance and quality. The recommended supplement dose of CGA for post-peaking laying hens is 400mg/kg.

## Supplementary Information

Supplementary Material 1: Table S1. Gene primer sequences for qPCR. Fig. S1. CGA supplementation improves expression of oxidative stress-related genes in liver and jejunum of post-peaking laying hens.

## Data Availability

The 16S rRNA sequencing data are available in the NCBI’s Sequence Read Archive (SRA) database under accession number PRJNA1240907. The RNA-seq data had been submitted to the SRA database with the accession number PRJNA1242568.

## Abbreviations

ACC-2: Acetyl-CoA carboxylase beta
ADEM: Average daily egg mass
AF: Atresia follicle
ADFI: Average daily feed intake
AKT: Protein kinase B
AMPK: AMP-activated protein kinase
APOB: Apolipoprotein B
APOVLDL-II: Apolipoprotein very low-density lipoprotein-II
BCL-2: B-cell lymphoma 2
BCL-XL: BCL2 like 1
CAS-9: Caspase-9
CAT: Catalase
CD: Crypt depth
CGA: Chlorogenic acid
CLDN-1: Claudin-1
CPT-1A: Carnitine palmitoyl transferase 1A
E2: Estradiol
ER-α: Estrogen receptor-α
ER-β: Estrogen receptor-β
FAO: Fatty acid oxidation
FCR: Feed conversion ratio
FSH: Follicle-stimulating hormone
FV: Fatty vacuole
GCLM: Recombinant glutamate cysteine ligase modifier subunit
GST: Glutathione S-transferase
IC: Inflammatory cell
IL-4: Interleukin-4
IL-6: Interleukin-6
IL-10: Interleukin-10
JAK-2: Janus kinase-2
JAK-3: Janus kinase-3
LH: Luteinizing hormone
LR: Laying rate
LW: Large white follicle
LY: Large yellow follicle
MDA: Malondialdehyde
MUC-2: Mucin-2
NAFLD: Non-alcoholic fatty liver disease
NF-κB: Nuclear factor kappa B
OCLN-1: Occludin-1
PROG: Progesterone
PCoA: Principal coordinate analysis
PF: Primary follicle
p-NF-κB: Phospho-nuclear factor kappa B
PI3K: Phosphatidylinositol 3-kinase
PLS-DA: Partial least squares discriminant analysis
p-PI3K: Phospho-phosphatidylinositol 3-kinase
PRDX-3: Peroxiredoxin-3
P53: Tumor protein p53
SF: Secondary follicle
SG: Stratum granulosum
SOD: Superoxide dismutase
SOD-1: Superoxide dismutase-1
SOD-2: Superoxide dismutase-2
STAT-3: Signal transducer and activator of transcription-3
SW: Small white follicle
SY: Small yellow follicle
T-AOC: Total antioxidant capacity
TE: Theca externa
TGF-β: Transforming growth factor-β
TI: Theca interna
TJ: Tight junction
TNF-α: Tumor necrosis factor-α
t-NF-κB: Total-nuclear factor kappa B
t-PI3K: Total-phosphatidylinositol 3-kinase
VH: Villus height
VTG-II: Vitellogenin-II
ZO-1: Zonula occludens-1
