# Supplementary material for: Integrated analysis of microbiome and transcriptome reveals the mechanisms underlying the chlorogenic acid-mediated attenuation of oxidative stress and systemic inflammatory responses via gut-liver axis in post-peaking laying hens
Source: J Anim Sci Biotechnol. 2025 Jun 6;16:82. doi: 10.1186/s40104-025-01216-7 (PMC12142991; doi:10.1186/s40104-025-01216-7)
Supplement: Supplementary file 1 — Supplementary Material 1: Table S1. Gene primer sequences for qPCR. Fig. S1. CGA supplementation improves expression of oxidative stress-related genes in liver and jejunum of post-peaking laying hens. [file 40104_2025_1216_MOESM1_ESM.docx]

**Supplementary Material**

**Table S1.** Genes primer sequences for qPCR

| Gene | Genbank Number | Primer Sequence (5' → 3') | Product Size |
| --- | --- | --- | --- |
| *β-actin* | NM_205518.2 | F:ACCGGACTGTTACCAACACC  R:CCTGAGTCAAGCGCCAAAAG | 116 bp |
| *KEAP-1* | MN416132.1 | F:TCAACTGGGTGCAGTACGAC  R:TCTGCGCCAGGTAATCCTTG | 162 bp |
| *NRF-2* | NM_205117.2 | F:CAGGGGTAGCAAGGTATGAGG  R:TTCCCAGTTCGGTGCAGAAG | 166 bp |
| *HO-1* | NM_205344.2 | F:CCACACAACGCTGAAAGCAT  R:GATGAAGTACAGGGACGCCG | 165 bp |
| *SOD1* | NM_205064.2 | F:TGACCTCGGCAATGTGACTG  R:CATGGTACGGCCAATGATGC | 103 bp |
| *SOD2* | NM_204211.2 | F:TGTTCAAGGATCAGGCTGGG  R:CCCAGCAATGGAATGAGACCT | 120 bp |
| *GST* | NM_001001777.2 | F:AATTTCCCCTCTTGCAGAGTT  R:TCACTCCACTTATCAGCAAACAG | 195 bp |
| *PRDX3* | XM_040674880.1 | F：GGCTCACACTGGAAATCGTGG  R：CACTCTTGCGCGGGGTATTT | 132 bp |
| *GCLM* | NM_001007953.2 | F:CTGAGTCACGGTGTCGCTC R:TTCTGAATGCAGTCCCGCAC | 186 bp |
| *OCLN-1* | NM_205128.1 | F:CTCAATCAGCTCAGCCGAC  R:TCTCCTGCTTCTTGCTTTGGTA | 130 bp |
| *CLDN-1* | NM_001013611.2 | F:GGTATGGCAACAGAGTGGCT  R:CAGCCAATGAAGAGGGCTGA | 91 bp |
| *ZO-1* | XM_040706827.2 | F:GTAAACCACTGCCTACACC  R:ATATCTTAACTCTACTTCGCACA | 90 bp |
| *MUC-2* | XM_040673077.2 | F:TGCCAGCCTTTTTATGCTCT  R:AGTGGCCATGGTTTCTTGTC | 80 bp |
| *VTG-II* | NM_001031276.2 | F:AACTACTCGATGCCCGCAAA  R:GCCCCATCTACCAGCAGTTT | 188 bp |
| *APOB* | NM_001044633.2 | F:GCAGCTTTGCTCATCGTGAC  R:AACGTCAGCAAATGTTGGGC | 119 bp |
| *APOVLDL-II* | NM_205483.3 | F:ACAGAGAACGTCGTGACTGG  R:CTGACCAGCTCTAGGGGACA | 92 bp |
| *ER-α* | NM_205183.2 | F:CTCTCACCCTTCA TCCATCACC  R:CCTCACAAGACCAGACCCCATA | 262 bp |
| *ER-β* | NM_001396358 | F:AAGAAGAGAACGCTGTGGGTAT  R:CTCGGTGAATGGTTTGCTAGGA | 208 bp |
| *JAK-3* | NM_204996.3 | F:GCCCCTGGACCTCTACCTAA  R:TCTTAGCAGAGACGTTGCCG | 143 bp |
| *PI3K* | [XM_046923808.1](https://www.ncbi.nlm.nih.gov/entrez/viewer.fcgi?db=nucleotide&id=2201819580) | F:AATCGTTGCCAAAGCTGCTG  R:TCTCACATACTGGTCGGGGT | 149 bp |
| *AKT-1* | [XM_046917865.1](https://www.ncbi.nlm.nih.gov/entrez/viewer.fcgi?db=nucleotide&id=2201800121) | F:GGCACATTCATTGGCTACAA  R:GGTCGTTCTGTCTTCATCAGC | 107 bp |
| *CAS-9* | [XM_046931415.1](https://www.ncbi.nlm.nih.gov/entrez/viewer.fcgi?db=nucleotide&id=2201773482) | F:GCGATTCCTTTCCAGGCTCC  R:CACGAGCCACTCACCTTGTC | 140 bp |
| *BCL-XL* | [XM_046930547.1](https://www.ncbi.nlm.nih.gov/entrez/viewer.fcgi?db=nucleotide&id=2201770701) | F:GGGTAGTGGTGGGACGCATTG  R:CGGTGAGGAGCCATTTGTTGA | 171 bp |
| BCL-2 | [NM_205339.3](https://www.ncbi.nlm.nih.gov/entrez/viewer.fcgi?db=nucleotide&id=2156850580) | F:ATCGTCGCCTTCTTCGAGTT  R:ATCCCATCCTCCGTTGTTCT | 150 bp |
| *P53* | [NM_205264.1](https://www.ncbi.nlm.nih.gov/entrez/viewer.fcgi?db=nucleotide&id=46048717) | F:GTCCCATCCACGGAGGATTAT  R:CCAGGCGGCAATAGACCTTA | 124 bp |
| *NF-ΚB* | [NM_205134.2](https://www.ncbi.nlm.nih.gov/entrez/viewer.fcgi?db=nucleotide&id=2117900867) | F:TCAACGCAGGACCTAAAGACAT  R:GCAGATAGCCAAGTTCAGGATG | 162 bp |
| *CPT-1A* | [XM_046918278.1](https://www.ncbi.nlm.nih.gov/entrez/viewer.fcgi?db=nucleotide&id=2201800931) | F:TCGTCTTGCCATGACTGGTG  R:GCTGTGGTGTCTGACTCGTT | 143 bp |
| *JAK-2* | NM_001030538.3 | F:GCACAAGCAGAGCATATCGC  R:TCGCCACTGTGCAAATAGGT | 95 bp |
| *STAT-3* | [NM_001398323.1](https://www.ncbi.nlm.nih.gov/entrez/viewer.fcgi?db=nucleotide&id=2164644537) | F:GACCAGATGCGAAGGGGTAT  R:CCACCAATGCAGGCAATTTGT | 131 bp |
| *AMPK* | [NM_001039603.2](https://www.ncbi.nlm.nih.gov/entrez/viewer.fcgi?db=nucleotide&id=2099396031) | F:TTCGCAGCCTTGACGTTGTA  R:TCCTTCTCATCAAGCCTTCCAT | 190 bp |
| *ACC-2* | [XM_040684650.1](https://www.ncbi.nlm.nih.gov/entrez/viewer.fcgi?db=nucleotide&id=2024460490) | F:CAAGAACGTCCGTGAGGAAC  R:TCTCTGCTGGGCAATGAGAA | 172 bp |

**
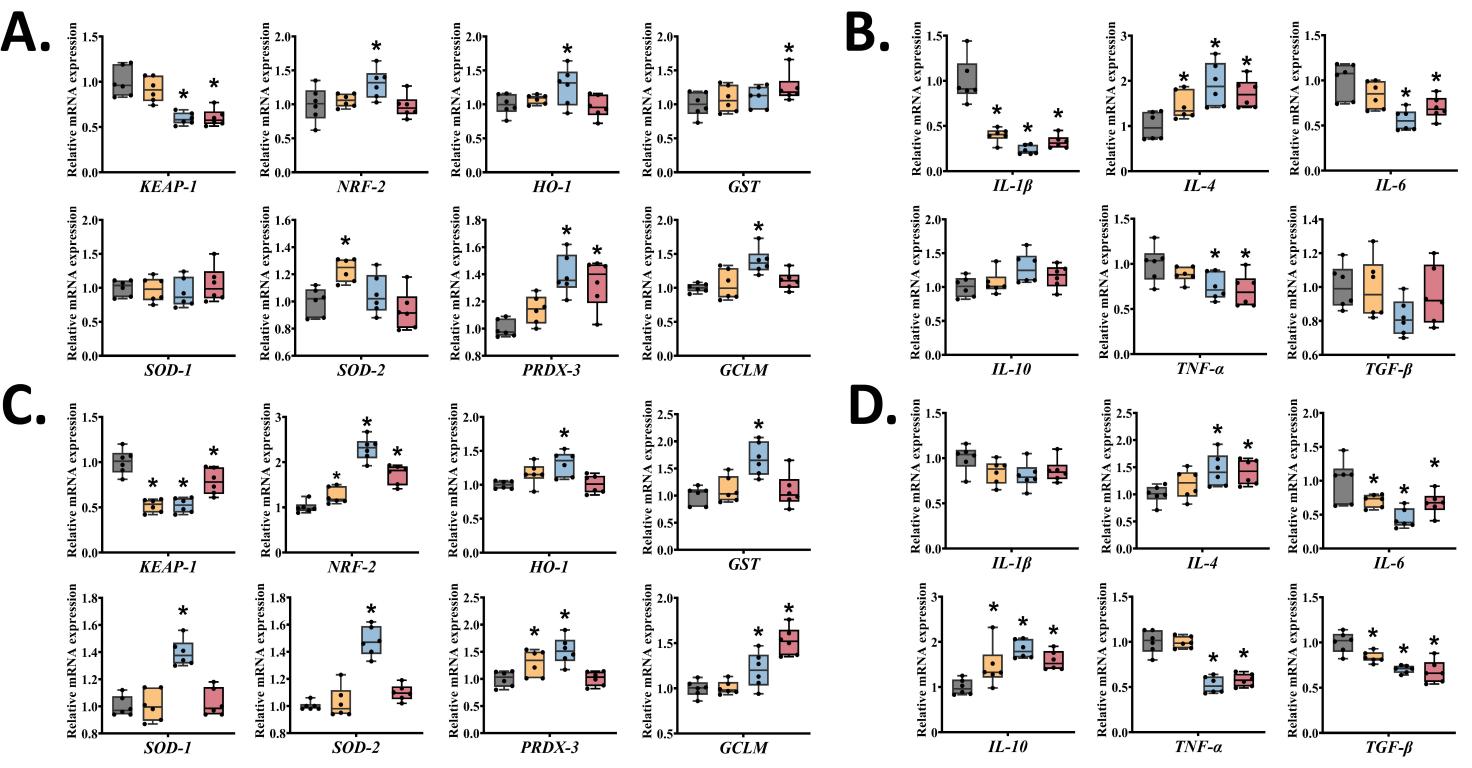
**

**Fig. S1.** CGA supplementation improved expression of oxidative stress-related genes in liver and jejunum of post-peaking laying hens (*n* = 6). (A-B) Relative mRNA expression levels of oxidative and inflammatory genes in liver tissue detected by qPCR. (C-D) Relative mRNA expression levels of oxidative and inflammatory genes in jejunum tissue detected by qPCR. All data were represented as mean ± SD. **P* < 0.05 compared to the CON group.
